# Distinctive molecular and biochemical characteristics of a glycoside hydrolase family 20 β-N-acetylglucosaminidase and salt tolerance

**DOI:** 10.1186/s12896-017-0358-1

**Published:** 2017-04-11

**Authors:** Junpei Zhou, Zhifeng Song, Rui Zhang, Rui Liu, Qian Wu, Junjun Li, Xianghua Tang, Bo Xu, Junmei Ding, Nanyu Han, Zunxi Huang

**Affiliations:** 1 Engineering Research Center of Sustainable Development and Utilization of Biomass Energy, Ministry of Education, Yunnan Normal University, Kunming, 650500 People’s Republic of China; 2 College of Life Sciences, Yunnan Normal University, No. 768 Juxian Street, Chenggong, Kunming, Yunnan 650500 People’s Republic of China; 3 Key Laboratory of Yunnan for Biomass Energy and Biotechnology of Environment, Yunnan Kunming, 650500 People’s Republic of China; 4 Key Laboratory of Enzyme Engineering, Yunnan Normal University, Kunming, 650500 People’s Republic of China

**Keywords:** β-N-Acetylglucosaminidase, Activity, N-Acetylglucosaminide tolerance, Salt tolerance, *Microbacterium*

## Abstract

**Background:**

Enzymatic degradation of chitin has attracted substantial attention because chitin is an abundant renewable natural resource, second only to lignocellulose, and because of the promising applications of N-acetylglucosamine in the bioethanol, food and pharmaceutical industries. However, the low activity and poor tolerance to salts and N-acetylglucosamine of most reported β-N-acetylglucosaminidases limit their applications. Mining for novel enzymes from new microorganisms is one way to address this problem.

**Results:**

A glycoside hydrolase family 20 (GH 20) β-N-acetylglucosaminidase (GlcNAcase) was identified from *Microbacterium* sp. HJ5 harboured in the saline soil of an abandoned salt mine and was expressed in *Escherichia coli*. The purified recombinant enzyme showed specific activities of 1773.1 ± 1.1 and 481.4 ± 2.3 μmol min^−1^ mg^−1^ towards *p*-nitrophenyl β-N-acetylglucosaminide and N,N'-diacetyl chitobiose, respectively, a *V*_max_ of 3097 ± 124 μmol min^−1^ mg^−1^ towards *p*-nitrophenyl β-N-acetylglucosaminide and a *K*_i_ of 14.59 mM for N-acetylglucosamine inhibition. Most metal ions and chemical reagents at final concentrations of 1.0 and 10.0 mM or 0.5 and 1.0% (v/v) had little or no effect (retaining 84.5 − 131.5% activity) on the enzyme activity. The enzyme can retain more than 53.6% activity and good stability in 3.0–20.0% (w/v) NaCl. Compared with most GlcNAcases, the activity of the enzyme is considerably higher and the tolerance to salts and N-acetylglucosamine is much better. Furthermore, the enzyme had higher proportions of aspartic acid, glutamic acid, alanine, glycine, random coils and negatively charged surfaces but lower proportions of cysteine, lysine, α-helices and positively charged surfaces than its homologs. These molecular characteristics were hypothesised as potential factors in the adaptation for salt tolerance and high activity of the GH 20 GlcNAcase.

**Conclusions:**

Biochemical characterization revealed that the GlcNAcase had novel salt–GlcNAc tolerance and high activity. These characteristics suggest that the enzyme has versatile potential in biotechnological applications, such as bioconversion of chitin waste and the processing of marine materials and saline foods. Molecular characterization provided an understanding of the molecular–function relationships for the salt tolerance and high activity of the GH 20 GlcNAcase.

**Electronic supplementary material:**

The online version of this article (doi:10.1186/s12896-017-0358-1) contains supplementary material, which is available to authorized users.

## Background

β-N-acetylglucosaminidases (GlcNAcases, EC 3.2.1.52) are glycoside hydrolases (GHs) that cut the O-glycosidic bonds formed by N-acetylglucosamine (GlcNAc) residues from the non-reducing terminals of oligosaccharides, such as chitooligosaccharides and muropeptides [1, 2]. Chitooligosaccharides can be produced from the degradation of chitin by hydrochloric acid [3] or chitinases (EC 3.2.1.14) [2]. Chitin is second only to lignocellulose in natural abundance, but more than 80,000 tons of chitin from marine sources per year is unutilized chitinous waste [2]. GlcNAcases hydrolyse chitooligosaccharides to produce GlcNAc, which can be further used for the production of bioethanol [4] and single cell protein [5], the treatment of ulcerative colitis and other gastrointestinal inflammation disorders [6] and pharmaceutical therapy for osteoarthritis [7] and tumours [8]. Muropeptides can be derived from the peptidoglycan of the cell wall [1] and flagella [9]. The hydrolysis of muropeptides by GlcNAcases indicates that the enzymes function in cell wall recycling [1] and flagellum assembly of bacteria [9]. Furthermore, GlcNAcases also exhibit many additional important biological functions and wide range of industrial applications, such as catabolism of ganglioside storage in Tay-Sachs disease [10], induction of chitinolytic enzymes [11] and synthesis of biologically important oligosaccharides [12, 13].

According to a homology comparison of amino acid sequences, GlcNAcases have been classified into GH 3, 20, 73 and 84 (http://www.cazy.org/). With the rapid development of genome sequencing technology, many GlcNAcases have been identified in the genomes of animal tissues, insects, plants, bacteria and fungi in recent years. GH 20 GlcNAcases are mainly found in microorganisms, especially bacteria (http://www.cazy.org/GH20.html). However, the molecular and biochemical characteristics of the majority of these GH 20 GlcNAcases have not been reported (http://www.cazy.org/GH20.html).

Yunnan Province is known as “the Kingdom of Nonferrous Metals”, “the Kingdom of Plants” and “the Kingdom of Animals” in China. In these natural habitats containing metal ions in high concentrations or special products from plants or animals, novel microorganisms and their enzymes may have to evolve, indicating biotechnological potential. Heijing is called the “town of salt” in Yunnan and has produced salts since ancient China. We previously sampled saline soil from an old mine in the town and reported novel enzymes from bacteria isolated from the soil [14–17]. One example is the GH 32 invertase InvHJ14, showing novel low-temperature and alkaline activity and sucrose tolerance [15]; another is the GH 27 α-galactosidase AgaAHJ8, showing novel salt − protease tolerance and transglycosylation activity, from a potential novel species of *Pontibacter* [16].

In this study, a novel GH 20 GlcNAcase, called HJ5Nag, was mined from the genome of *Microbacterium* sp. HJ5—a new isolate from the saline soil. The purified recombinant HJ5Nag (rHJ5Nag), which was expressed in *E. coli*, showed better tolerance to salts and GlcNAc and higher activity than most GlcNAcases reported in the literature, including the GH 3 GlcNAcase we previously reported [18]. Potential molecular adaptations for salt tolerance and the high activity of the enzyme were presumed.

## Methods

### Strains, vectors and reagents

Strain HJ5 was isolated from the saline soil of an old salt mine located in Heijing Town. The details of the strain isolation and identification were described in our previous study [14]. The pure culture was deposited in the Strains Collection of the Yunnan Institute of Microbiology under registration no. YMF 4.00007. *E. coli* BL21 (DE3) was purchased from TransGen (Beijing, China) and was used for gene expression.

The following vectors and reagents were used: plasmid isolation and genomic DNA kits (Tiangen, Beijing, China); dNTPs and DNA polymerases (TaKaRa, Otsu, Japan); isopropyl-β-d-1-thiogalactopyranoside (IPTG; Amresco, Solon, OH, USA); Ni^2+^-NTA agarose (Qiagen, Valencia, CA, USA); Qubit protein assay kit (Invitrogen, Carlsbad, CA, USA); *pEASY*-E2 vector (TransGen); N,N'-diacetyl chitobiose (GlcNAc_2_), N,N',N'',N'''-tetraacetyl chitotetraose (GlcNAc_4_), *p*-nitrophenyl β-N-acetylgalactosaminide (*p*NPGalNAc), and *p*-nitrophenyl β-d-glucopyranoside (*p*NPGlc) (J&K Scientific Ltd., Beijing, China); peptidoglycan from *Bacillus subtilis* (Ekear, Shanghai, China); *p*-nitrophenol (*p*NP), *p*-nitrophenyl-β-d-xylopyranoside (*p*NPXyl), *p*-nitrophenyl α-d-galactopyranoside (*p*NPGal), *p*-nitrophenyl-α-l-arabinofuranoside (*p*NPAra), *p*-nitrophenyl β-N-acetylglucosaminide (*p*NPGlcNAc), mutanolysin from *Streptomyces globisporus* ATCC 21553, chitin and chitosan (Sigma − Aldrich, St. Louis, MO, USA); and silica gel G plates (Haiyang, Qingdao, China). All other chemicals were of analytical grade.

### Gene cloning and sequence analysis

The GlcNAcase-encoding gene, designated *hj5Nag*, was obtained by genome sequencing performed on a Miseq sequencer (Illumina) in our lab. The HJ5 genome sequencing details were described in our previous study [19].

Open reading frames from the draft genome of HJ5 were predicted using the tools described in our previous study [17]. BLASTN and BLASTP (http://www.ncbi.nlm.nih.gov/BLAST/) online tools were used to search homologous sequences and to calculate the identity values. SignalP (http://www.cbs.dtu.dk/services/SignalP/) and InterPro (http://www.ebi.ac.uk/interpro/) online tools were used to predict the signal peptide and protein domain, respectively. Vector NTI 10.3 (InforMax, Gaithersburg, MD, USA) software was used to calculate frequencies of the amino acid residues. Multiple sequences and structures alignments were generated by ESPript [20].

### Structure analysis

The homology model of HJ5Nag was predicted using the SwissModel platform (http://swissmodel.expasy.org/). The proportions of secondary structures were analysed by VADAR [21]. The charge distributions on the surfaces of various GlcNAcases were calculated using Discovery Studio 2.5 software (Accelrys, San Diego, CA, USA).

### Heterologous expression of HJ5Nag

The coding sequence of HJ5Nag was amplified by PCR using the primer set r*hj5Nag*EF (5'-GGGTGCAGCCCCGCCGC-3') and r*hj5Nag*ER (5'-CTCGGTGGCCCAGTCGATCTCGC-3'). The resulting PCR product was ligated to *pEASY*-E2 vector, which has a single 3′-T overhang at the insertion site according to the manufacturer. *E. coli* BL21 (DE3) competent cells were transformed with the plasmid for recombinant enzyme expression. A positive transformant harbouring the recombinant plasmid was confirmed by DNA sequencing performed by Tsingke (Beijing, China). A seed culture of the positive transformant was grown overnight at 37 °C and then inoculated in 1:100 dilutions into fresh Luria–Bertani medium with the addition of 100 μg mL^−1^ ampicillin. Upon reaching an OD_600 nm_ of approximately 0.7, IPTG at a final concentration of 0.25 mM was added to the culture to induce enzyme expression at 20 °C for approximately 20 h.

### Purification and identification of recombinant GlcNAcase

Cultures containing positive transformant cells were centrifuged and resuspended in ice-cold buffer A containing 20 mM Tris–HCl, 0.5 M NaCl, and 10% (w/v) glycerol (pH 7.2). The cells were disrupted by ultrasonication on ice with 100 short bursts of 4 s each at a power output of 150 W. After removing cell debris by centrifugation, the supernatant was loaded onto Ni^2+^-NTA agarose gel columns to bond the recombinant enzyme. The target recombinant enzyme was eluted with a linear imidazole gradient of 20–500 mM in buffer A. The protein concentration was determined using a Qubit protein assay kit using a Qubit 2.0 fluorometer (Invitrogen).

Sodium dodecyl sulphate–polyacrylamide gel electrophoresis (SDS–PAGE) was performed to analyse the expression of the recombinant enzyme and the purity of the eluted fractions. The molecular masses of internal peptides from the single band present in the SDS–PAGE gel were analysed via matrix-assisted laser desorption/ionization time-of-flight mass spectrometry (MALDI–TOF MS) and were compared with the molecular masses of the internal peptides from rHJ5Nag.

### Enzyme assay and substrate specificity

An enzyme assay of purified rHJ5Nag towards various substrates was performed spectrophotometrically using the *p*NP and 3,5-dinitrosalicylic acid (DNS) methods, as described in our previous study [18]. The *p*NP method was applied to determine the activity of rHJ5Nag towards substrates *p*NPXyl, *p*NPGal, *p*NPGlc, *p*NPAra, *p*NPGlcNAc and *p*NPGalNAc. The DNS method was used for the substrates GlcNAc_2_, GlcNAc_4_, colloidal chitin, chitosan, peptidoglycan and muropeptides (degradation products of peptidoglycan by mutanolysin treatment).

### Biochemical characterization

The *p*NP method using *p*NPGlcNAc as substrate was applied to determine the biochemical characteristics of purified rHJ5Nag, unless otherwise noted. McIlvaine buffer was used for pH 5.0–8.0, and 0.1 M glycine–NaOH was used for pH 9.0–12.0.

The pH-dependent activity of rHJ5Nag was determined at 37 °C, with pH values ranging from 5.0 to 9.0. The pH-dependent stability of rHJ5Nag was determined by measuring the residual enzyme activity after incubating the enzyme at pH 5.0–12.0 for 1 h.

The temperature-dependent activity of rHJ5Nag was examined at pH 6.0 and temperatures from 0 to 60 °C. The thermostability of rHJ5Nag was determined by measuring the residual activity of the enzyme after pre-incubation at 30, 45 and 50 °C and pH 6.0 for 1–60 min.

To determine the effects of various metal ions and chemical reagents on purified rHJ5Nag, the enzyme activities were measured after the addition of different metal ions and chemical reagents to the reaction mixture. The metal ions and chemical reagents included 1.0 or 10.0 mM (final concentration) KCl, AgNO_3_, CaCl_2_, CoCl_2_, HgCl_2_, Pb(CH_3_COO)_2_, MgSO_4_, NiSO_4_, CuSO_4_, MnSO_4_, ZnSO_4_, FeCl_3_, EDTA, β-mercaptoethanol and SDS, and 0.5 or 1.0% (v/v) Tween 80 and Triton X-100. The absorption was measured after removing the precipitate by centrifugation when AgNO_3_, HgCl_2_ or SDS was added to the reaction mixture. Furthermore, the enzyme activity in 1.0–30.0% (w/v) NaCl was also examined, and the enzyme stability in NaCl was determined by measuring the residual enzyme activity after pre-incubating the enzyme in 3.0–30.0% (w/v) NaCl for 1 h.

The *K*_m_, *V*_max_ and *k*_cat_ values of purified rHJ5Nag were analysed using 0.1–1.2 mM *p*NPGlcNAc as substrate at 45 °C and pH 6.0. A nonlinear regression Michaelis − Menten fit was applied to analyse the data using GraphPad Prism (GraphPad Software, San Diego, CA, USA). The extent of GlcNAc inhibition on rHJ5Nag activity was determined adding 5.0 and 10.0 mM GlcNAc to the reaction solution. Then, the β-N-acetylglucosaminidase activity was determined according to the aforementioned kinetic assay. The inhibition constant (*K*_i_) was calculated using GraphPad Prism using the nonlinear mixed-model inhibition equation described detailedly in a previous paper [22].

### Hydrolytic property of purified rHJ5Nag

The hydrolysis mixture included 160 μL of 0.5% (w/v) GlcNAc_2_ or GlcNAc_4_ and approximately 0.1 units of purified rHJ5Nag. After incubating the mixture in McIlvaine buffer (pH 6.0) at 37 °C for 6 h, the hydrolysis products were analysed by thin-layer chromatography (TLC), as previously described [17].

### Synergistic action

Commercial chitinase from *Streptomyces griseus*, designated CtnSg, was purchased from YuanYe Bio-Technology (Shanghai, China). The reaction mixture contained colloidal chitin (0.5%, w/v) and either CtnSg (0.01 U mL^−1^ reaction system), rHJ5Nag (0.5 U mL^−1^ reaction system) or both enzymes and was incubated for 120 min. In the time-course study, the reaction mixture containing 0.5% (w/v) colloidal chitin and CtnSg alone was incubated for 30, 60 or 90 min, rHJ5Nag was then added to the reaction and incubated for 90, 60 or 30 min, respectively. According to the manufacturer’s instructions for CtnSg and the biochemical characteristics of rHJ5Nag, degradation of colloidal chitin was performed at 25 °C and pH 6.0 (McIlvaine buffer). The DNS method was used to measure the amount of reducing sugars released from the colloidal chitin. A reaction containing heat-inactivated enzyme (incubating in a boiling water bath for 10 min) was used as a control. The degree of synergy was calculated using the following equations:

$$ D S=\frac{R{S}_{rHJ5 Nag CtnSg}}{R{S}_{rHJ5 Nag}+ R{S}_{CtnSg}} $$

where *DS* is the degree of synergy, *RS*_rHJ5NagCtnSg_ is the amount of reducing sugars released from simultaneous or sequential enzyme reactions, and *RS*_rHJ5Nag_ and *RS*_CtnSg_ are the amounts of reducing sugars released from individual enzyme reactions.

### Accession numbers

The accession numbers of *hj5Nag* and *Microbacterium* sp. HJ5 16S rDNA in GenBank are KX400857 and KX400858, respectively.

## Results

### Strain identification

Based on the results of a BLASTN search, the nucleotide identity was 98.8% between the partial 16S rDNA sequence from HJ5 (1375 bp) and the 16S rDNA sequences from *Microbacterium flavescens* (accession no. HQ530520), *Microbacterium takaoensis* (AB201047) and *Microbacterium trichothecenolyticum* (KU179351). Phylogenetic analysis also placed HJ5 in the *Microbacterium* cluster but not in the clusters grouped by the species of other genera of *Microbacteriaceae* (Additional file 1: Figure S1). Therefore, HJ5 belonged to the genus *Microbacterium*.

### Gene cloning and sequence analysis

Sequence data of approximately 606.6 Mbp was generated by genome sequencing for HJ5, and these data yielded a draft genomic DNA sequence of approximately 3.6 Mbp after sequence assembly. The analysis of the draft genome predicted an open reading frame designated gene *hj5Nag. hj5Nag* has a length of 1608 bp and encodes the 535-residue GlcNAcase HJ5Nag, with a predicted molecular mass of 55.9 kDa.

The alanine (A) and glycine (G) frequencies of the GlcNAcase were 16.3 and 10.8%, respectively, both of which are the highest values among the GlcNAcases shown in Table 1. The frequency of acidic aspartic acid (D) and glutamic acid (E) of the GlcNAcase was 13.5%, which was the third highest (Table 1). However, frequencies of cysteine (C) and lysine (K) in the GlcNAcase were only 0.2 and 1.1%, respectively, both of which were the second lowest (Table 1). Therefore, the ratio of the total frequency of D, E, A and G to the total frequency of C and K of HJ5Nag (31.2) was the highest among the GlcNAcases shown in Table 1.

**Table 1:** Amino acid residue frequencies of the experimentally characterized GH 20 GlcNAcases

GlcNAcase (PDB ID or accession no.)	Frequency (%)						Organism
	D & E	A	G	C	K	DEAG/CK	
ADJ68332	14.3	7.7	6.9	0.0	4.7	6.1	*Vibrio harveyi* [71]
AAC44672	13.7	8.2	5.2	1.6	3.8	5.0	*Vibrio furnissii* [37]
KX400857	13.5	16.3	10.8	0.2	1.1	31.2	*Microbacterium* sp. (This study)
ADJ68333	13.5	7.6	5.6	1.9	4.5	4.2	*V. harveyi* [71]
3RCN (WP_014923268)	13.4	11.2	10.1	0.2	1.7	18.3	*Arthrobacter aurescens* (Unpublished)
3NSM (ABI81756)	13.3	7.5	5.9	2.0	6.1	3.3	*Ostrinia furnacalis* [72]
ABA27426	13.0	4.5	6.5	1.3	5.1	3.8	*Spodoptera frugiperda* [70]
ABA27427	13.0	4.5	6.5	1.3	5.1	3.8	*S. frugiperda* [70]
4PYS	12.9	6.1	7.5	1.6	6.1	3.4	*Bacteroides fragilis* (Unpublished)
BAD00143	12.9	9.5	7.6	0.7	7.1	3.8	*Aeromonas hydrophila* [73]
CAH55822	12.9	9.7	7.6	0.7	7.1	3.9	*Aeromonas caviae* [48]
1QBA (AAB03808)	12.8	9.9	8.4	0.7	6.9	4.1	*Serratia marcescens* [23]
BAC76622	12.8	14.4	8.7	0.5	0.7	29.9	*Streptomyces thermoviolaceus* [42]
AAQ05800	12.7	12.1	8.9	0.0	1.2	28.1	*Cellulomonas fimi* [45]
1 M01 (AAC38798)	12.5	11.9	10.5	0.4	3.6	8.7	*Streptomyces plicatus* [74]
BAC41255	12.5	8.0	5.7	1.2	3.0	6.2	*Aspergillus oryzae* [12]
AKC34129	12.3	9.7	7.1	0.4	7.1	3.9	Uncultured organism [44]
BAA92145	12.2	10.1	9.7	0.7	4.3	6.4	*Aeromonas* sp. [75]
2GJX (AAD13932)	11.9	4.9	6.4	1.5	3.6	4.5	*Homo sapiens* [24]
BAF76001	11.8	9.1	8.3	1.8	2.4	7.0	*A. hydrophila* [76]
CAD10500	11.5	6.9	5.7	1.8	6.2	3.0	*Entamoeba histolytica* [77]
BAM42836	11.2	11.2	6.5	1.1	1.6	10.7	*Lentinula edodes* [78]
AKC34128	10.7	14.4	9.1	0.4	1.6	17.1	Uncultured organism [44]
3GH4 (BAI63641)	10.5	8.0	8.8	0.4	5.1	5.0	*Paenibacillus* sp. [25]
BAA88762	10.4	12.9	10.0	0.4	2.4	11.9	*S. thermoviolaceus* [46]
BAA06136	10.4	10.0	5.3	1.2	6.0	3.6	*Pseudoalteromonas piscicida* (previous *Alteromonas* sp.) [79]
BAB84321	10.4	8.3	5.1	0.4	4.9	4.5	*P. piscicida* (previous *Alteromonas* sp.) [80]
CAE46968	10.3	4.3	5.1	1.4	6.4	2.5	*E. histolytica* [77]
BAA76352	10.1	15.2	5.5	0.0	7.2	4.3	*Lactobacillus casei* [41]
EGR50812	8.9	10.0	6.4	1.0	3.4	5.8	*Trichoderma reesei* [49]

**Fig. 1:**
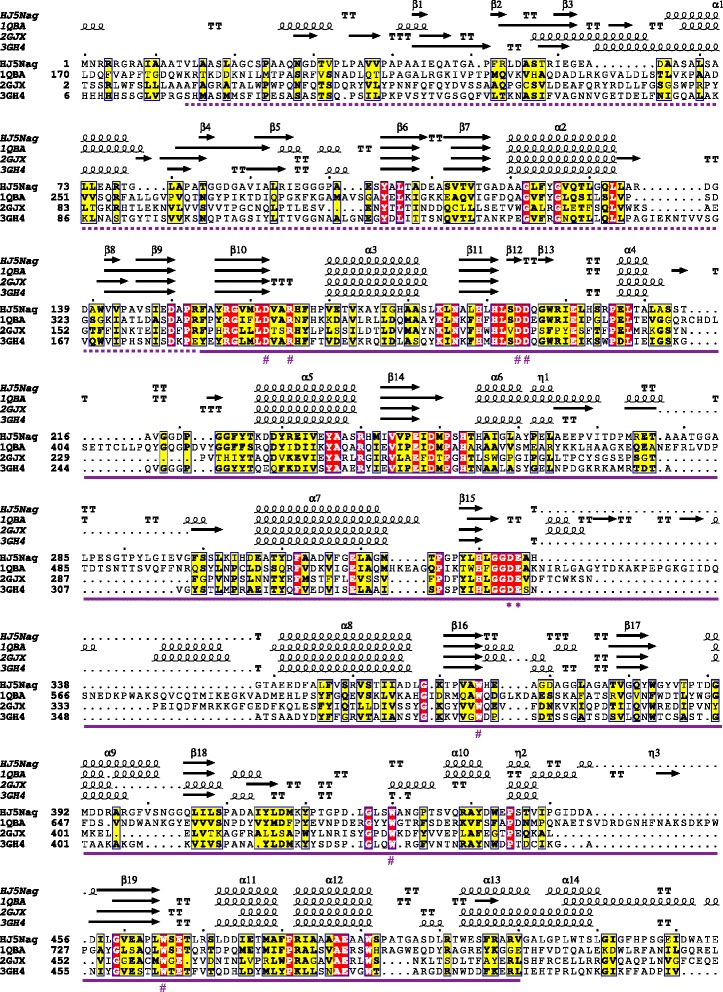
Partial amino acid sequences and structures alignments of HJ5Nag with GH 20 GlcNAcases. Sequence names are shown with PDB IDs (except HJ5Nag) as follows: 1QBA (accession no. AAB03808), the GlcNAcase from *S. marcescens* [23]; 2GJX (accession no. AAD13932), the GlcNAcase from *H. sapiens* [24]; and 3GH4 (accession no. BAI63641), the GlcNAcase from *Paenibacillus* sp. [25]. *Asterisks* and *number signs* show the putative catalytic residues and ligand-binding residues, respectively. *Dotted purple line* shows the N-terminal domain of bacterial GlcNAcases (domain signature: IPR015882). *Solid purple line* shows the catalytic domain of GH 20 GlcNAcases (domain signature: IPR015883)

The predicted signal peptide of HJ5Nag is cleaved between A25 and A26 to yield a mature polypeptide consisting of the typical N-terminal domain of bacterial GlcNAcases (from V14 to R153; domain signature: IPR015882) and the catalytic domain of GH 20 GlcNAcases (from F154 to V510; IPR015883; Fig. 1, Additional file 1: Figure S2). A BLASTP search for HJ5Nag showed that the enzyme was most similar to multiple putative GH 20 GlcNAcases annotated based on genome analysis. Among these putative GH 20 members, the enzyme from *Microbacterium* sp. Root180 (accession no. WP_056119055) shared the highest identity of 78.9% with HJ5Nag. Based on the alignment of HJ5Nag with other experimentally characterized GlcNAcases (Fig. 1) [23–25], residue D334 probably functioned as the catalytic nucleophile/base, and E335 probably functioned as the catalytic proton donor/acceptor. In addition, residues D162, R165, D194, D195, W365, W430 and W465 might play an important role in binding the GlcNAcase ligand (Fig. 1) [23–25].

### Structure analysis

**Fig. 2:**
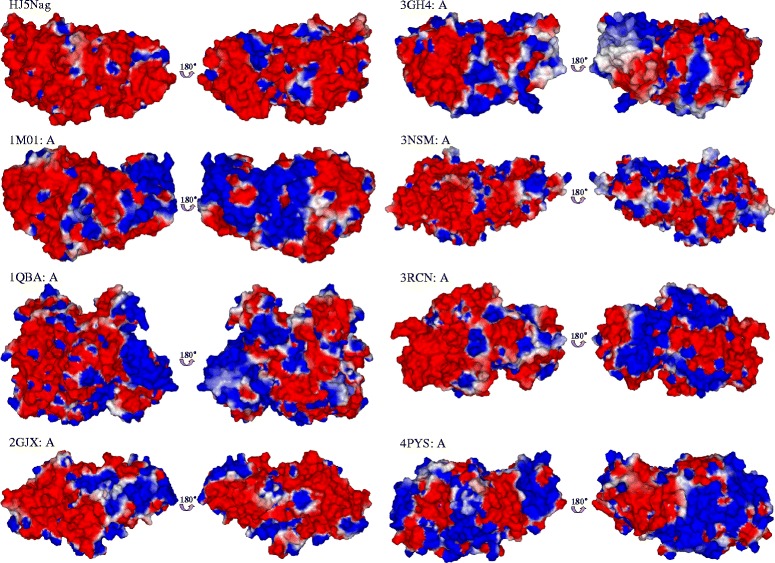
Charge distributions on the surfaces of the GH 20 GlcNAcases. The A-chain of GlcNAcase is used. PDB IDs are presented as the names of GlcNAcases (except HJ5Nag) as follows: 1 M01, the GlcNAcase from *S. plicatus* [74]; 1QBA, the GlcNAcase from *S. marcescens* [23]; 2GJX, the GlcNAcase from *H. sapiens* [24]; 3GH4, the GlcNAcase from *Paenibacillus* sp. [25]; 3NSM, the GlcNAcase from *O. furnacalis* [72]; 3RCN, the GlcNAcase from *A. aurescens*; 4PYS, the GlcNAcase from *B. fragilis*. The charge distribution on the surface was calculated at pH 7.0. Positive charges are depicted in *blue* and negative charges in *red*

The model of HJ5Nag showed a GMQE score of 0.72 and a sequence identity of 48.9% when the template was the crystal structure of the GH 20 GlcNAcase from *Streptomyces plicatus* (PDB ID: 1 M01). The homology model of HJ5Nag and the crystal structures of 7 GlcNAcases were used to calculate the proportions of secondary structures and the charge distributions on the surfaces. Among these GlcNAcases, the proportions of amino acids used to build the α-helix and β-sheet structures of HJ5Nag were the second lowest, while the proportion of amino acids used to build random coil structures and the ratio of random coils to α-helices of HJ5Nag were both the highest (Table 2). As shown in Fig. 2, almost the whole surface of HJ5Nag was negatively charged. Compared with other GlcNAcases, HJ5Nag had a much larger negatively charged surface (Fig. 2).

**Table 2:** Proportions of secondary structures of GH 20 GlcNAcases

GlcNAcase (PDB ID)	α-Helix	β-Sheet	Coil	Coil/α-Helix
2GJX [24]	176 (35.8%)	133 (27.0%)	183 (37.2%)	1.04
3RCN (Unpublished)	185 (35.2%)	146 (27.8%)	194 (37.0%)	1.05
1 M01 [74]	170 (34.1%)	121 (24.2%)	208 (41.7%)	1.22
3NSM [72]	193 (33.7%)	162 (28.3%)	217 (37.9%)	1.12
4PYS (Unpublished)	165 (33.5%)	134 (27.2%)	193 (39.2%)	1.17
3GH4 [25]	165 (32.5%)	137 (27.0%)	205 (40.4%)	1.24
KX400857 (This study)	152 (30.2%)	126 (25.0%)	226 (44.8%)	1.49
1QBA [23]	232 (27.0%)	282 (32.9%)	344 (40.1%)	1.48

### Expression, purification and identification of rHJ5Nag

As shown by an SDS–PAGE gel (Additional file 1: Figure S3), the recombinant GlcNAcase was successfully expressed in *E. coli* BL21 (DE3) and purified by Ni^2+^-NTA affinity chromatography. The MALDI–TOF MS spectrum of the purified band further confirmed that the purified product was rHJ5Nag (Additional file 1: Figure S4).

### Biochemical characterization

At pH 6.0 and 45 °C, purified rHJ5Nag showed specific activities of 1773.1 ± 1.1 (Table 3), 146.0 ± 3.1, 481.4 ± 2.3 and 445.0 ± 1.7 μmol min^−1^ mg^−1^ towards substrates of *p*NPGlcNAc, *p*NPGalNAc, GlcNAc_2_ and GlcNAc_4_, respectively. However, no rHJ5Nag activity was detected towards substrates of *p*NPXyl, *p*NPAra, *p*NPGlc, *p*NPGal, peptidoglycan, muropeptides, colloidal chitin or chitosan. Thus, rHJ5Nag is a GlcNAcase that can hydrolyse chitooligosaccharides but cannot participate in cell wall turnover or recycling of bacteria.

**Table 3:** Activities of the experimentally characterized GlcNAcases

GlcNAcase	GH Family	Specific activity^a^	*V*_max_^a^	Organism
-	84	1.2	212	*Penicillium chrysogenum* [81]
NagA	20	1926.0	-	*A. oryzae* [12]
HJ5Nag	20	1773.1	3097	*Microbacterium* sp. (This study)
-	20	913.6	-	*P. piscicida* (previous *Alteromonas* sp.) [79]
Nag1	20	319.9	-	*T. reesei* [49]
ExoI	20	200	270	*V. furnissii* [37]
-	20	73.3	-	*L. casei* [41]
Sfhex	20		68.4	*S. frugiperda* [70]
NagC	20	44.7	-	*S. thermoviolaceus* [42]
NAG20A	20	30.0	115	*A. hydrophila* [73]
*Vh*Nag2	20	19.4	-	*V. harveyi* [82]
-	20	3.8	3.8	*E. histolytica* [77]
Hex1	20	-	212	*Paenibacillus* sp. [25]
Hex2	20	-	150	*Paenibacillus* sp. [25]
NagA	3	64.4	24.8	*S. thermoviolaceus* [83]
RmNag	3	21.2	49.3	*Rhizomucor miehei* [38]
Nag3HWLB1	3	19.4	19.9	*Sphingobacterium* sp. [18]
NahA	3	8.8	-	*Symbiobacterium thermophilum* [47]
ExoII	3	0.7	1.1	*V. furnissii* [68]
NagA	3	-	0.3	*Thermotoga maritima* [84]
CbsA	3	-	0.3	*Thermotoga neapolitana* [84]
-	-	521.5	-	*Enterobacter* sp. [85]
-	-	67.2	-	*Vibrio alginolyticus* [65]
-	-	40.5	-	*Talaromyces emersonii* [86]
-	-	27	2.0	*Streptomyces cerradoensis* [40]
NagA	-	5.3	245	*Pseudomonas fluorescens* [3]
	-	0.03	-	*Trichinella spiralis* [64]

^a^These values (μmol min^−1^ mg^−1^) were determined using *p*NPGlcNAc as substrate

**Fig. 3:**
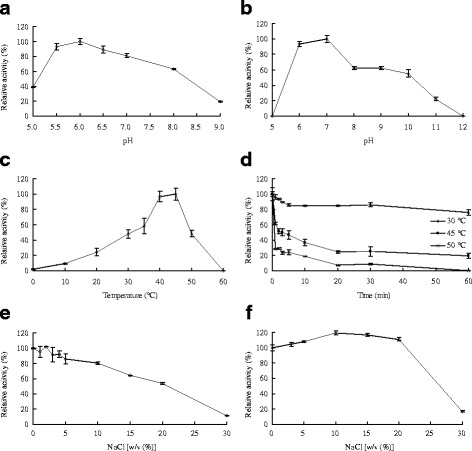
Enzymatic properties of purified rHJ5Nag. **a** pH-dependent activity profiles. **b** pH-dependent stability profiles. **c** Temperature-dependent activity profiles. **d** Thermostability assay. **e** Effect of NaCl on rHJ5Nag activity. **f** Stability of rHJ5Nag in NaCl. *Error bars* represent the means ± SD (*n* = 3)

Purified rHJ5Nag had activity in the range of pH 5.5–9.0 and 10 − 50 °C, with apparent optima at pH 6.0 and 45 °C (Fig. 3a, c). The enzyme exhibited good stability at 30 °C and maintained more than 55% of its initial activity after incubation in buffers ranging from pH 6.0 to 10.0 for 1 h (Fig. 3b, d).

Purified rHJ5Nag exhibited good salt tolerance. Although the activity of purified rHJ5Nag was completely inhibited by AgNO_3_, HgCl_2_ and SDS, the addition of most metal ions and chemical reagents at final concentrations of 1.0 and 10.0 mM or 0.5 and 1.0% (v/v) showed little to no effect (retaining 84.5 − 131.5% activity) on the enzyme activity (Table 4). Furthermore, the enzyme retained more than 80% activity in the presence of 1.0–10.0% (w/v) NaCl and even 53.6% activity in 20.0% (w/v) NaCl (Fig. 3e). The enzyme retained approximately 100% of its initial activity after incubation in 3.0–20.0% (w/v) NaCl for 60 min (Fig. 3f).

**Table 4:** Effects of metal ions and chemical reagents on the activity of purified rHJ5Nag

Reagent	Relative activity (%)^a^		Reagent	Relative activity (%)^a^	
	1.0 mM	10.0 mM		1.0 mM	10.0 mM
None	100.0 ± 0.4	100.0 ± 2.7	MnSO_4_	100.9 ± 2.6	99.6 ± 2.4
FeCl_3_	117.4 ± 1.8	103.1 ± 0.0	ZnSO_4_	96.8 ± 3.1	84.5 ± 9.9
MgSO_4_	113.8 ± 5.8	99.3 ± 3.4	AgNO_3_	0.0	0.0
CaCl_2_	108.7 ± 5.4	96.6 ± 3.5	HgCl_2_	0.0	0.0
KCl	105.4 ± 4.8	101.4 ± 1.3	Tween 80	131.5 ± 1.7^b^	108.1 ± 2.1^c^
CoCl_2_	103.7 ± 3.1	100.4 ± 1.7	EDTA	106.7 ± 4.5	99.5 ± 2.1
NiSO_4_	101.7 ± 8.7	95.0 ± 0.7	β-Mercaptoethanol	106.1 ± 1.2	100.1 ± 0.6
CuSO_4_	101.4 ± 4.8	88.6 ± 5.5	Triton X-100	100.5 ± 4.7^b^	93.9 ± 5.2^c^
Pb(CH_3_COO)_2_	101.0 ± 5.8	106.7 ± 3.7	SDS	0.0	0.0

^a^Values represent the means ± SD (*n* = 3) relative to the untreated control sample

^b^Final concentration: 0.5% (w/v)

^c^Final concentration: 1.0% (w/v)

**Fig. 4:**
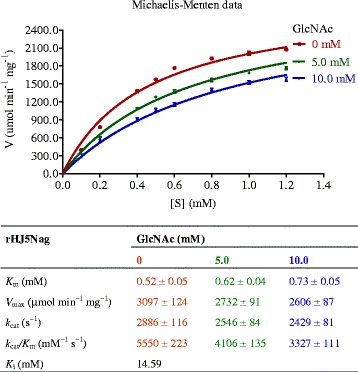
Kinetic characterization of purified rHJ5Nag. *Error bars* represent the means ± SD (*n* = 3)

Without the addition of GlcNAc, the *K*_m_, *V*_max_, *k*_cat_ and *k*_cat_/*K*_m_ of purified rHJ5Nag towards *p*NPGlcNAc were 0.52 ± 0.05 mM, 3097 ± 124 μmol min^−1^ mg^−1^, 2886 ± 116 s^−1^ and 5550 ± 223 mM^−1^ s^−1^, respectively (Table 3; Fig. 4). When 5.0 and 10.0 mM GlcNAc was added to the reaction solution, the *V*_max_ of purified rHJ5Nag towards *p*NPGlcNAc decreased to 2732 ± 91 and 2606 ± 87 μmol min^−1^ mg^−1^, respectively, the *k*_cat_ decreased to 2546 ± 84 and 2429 ± 81 s^−1^, respectively, and the *k*_cat_/*K*_m_ decreased to 4106 ± 135 and 3327 ± 111 mM^−1^ s^−1^, respectively (Fig. 4). However, the *K*_m_ of purified rHJ5Nag towards *p*NPGlcNAc increased to 0.62 ± 0.04 and 0.73 ± 0.05 mM in the presence of 5.0 and 10.0 mM GlcNAc, respectively (Fig. 4). Furthermore, the value of the parameter Alpha in the mixed-model inhibition equation was 3.412 based on the analysis of kinetic data using GraphPad Prism. These results indicate that GlcNAc can inhibit the activity of rHJ5Nag via a mixture of competitive and noncompetitive inhibition. As a result, the enzyme was inhibited by GlcNAc, with a *K*_i_ of 14.59 mM.

### Hydrolytic properties of purified rHJ5Nag

**Fig. 5:**
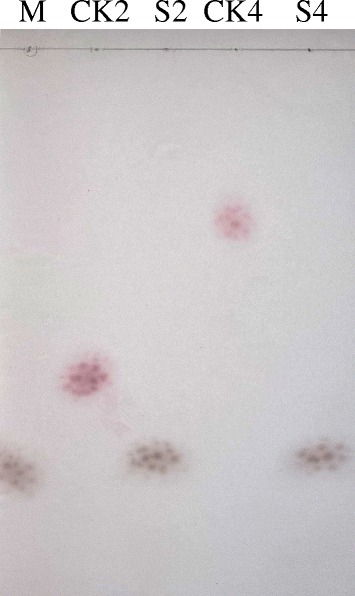
TLC analysis of the hydrolytic product. Lanes: M, 0.5% (w/v) GlcNAc; CK2 and CK4, inactivated rHJ5Nag (incubating in a boiling water bath for 10 min) with 0.5% (w/v) GlcNAc_2_ and GlcNAc_4_, respectively; S2 and S4, hydrolysis of 0.5% (w/v) GlcNAc_2_ and GlcNAc_4_ by purified rHJ5Nag, respectively

As shown in Fig. 5, GlcNAc was the end product of GlcNAc_2_ and GlcNAc_4_ hydrolysed by purified rHJ5Nag.

### Synergistic action

Although purified rHJ5Nag cannot hydrolyse colloidal chitin alone, simultaneous addition of CtnSg and rHJ5Nag improved the degradation of colloidal chitin by 2.02-fold (Table 5). Sequential addition of the two enzymes improved the degradation of colloidal chitin by 1.98-fold, 1.72-fold and 1.29-fold when rHJ5Nag was added and incubated for 90, 60 and 30 min, respectively (Table 5). Furthermore, these results indicate that the synergistic effect benefits from the earlier addition of rHJ5Nag.

**Table 5:** Synergy between the commercial chitinase CtnSg and rHJ5Nag for the degradation of colloidal chitin

Order of enzyme addition and reaction time				Reducing sugars (μmol)	Degree of synergy^a^
First enzyme	Time (min)	Second enzyme	Time (min)		
CtnSg	120	No	0	0.28	1.00
rHJ5Nag	120	No	0	ND^b^	-
CtnSg & rHJ5Nag	120	No	0	0.57	2.02
CtnSg	30	rHJ5Nag	90	0.55	1.98
CtnSg	60	rHJ5Nag	60	0.48	1.72
CtnSg	90	rHJ5Nag	30	0.36	1.29

^a^The degree of synergy was calculated using the following equations:

$$ D S=\frac{R{S}_{rHJ5 Nag CtnSg}}{R{S}_{rHJ5 Nag}+ R{S}_{CtnSg}} $$

where *DS* is the degree of synergy, *RS*_rHJ5NagCtnSg_ is the amount of reducing sugars released from simultaneous or sequential enzyme reactions, and *RS*_rHJ5Nag_ and *RS*_CtnSg_ are the amounts of reducing sugars released from individual enzyme reactions

^b^Not detected

## Discussion

Microorganisms isolated from extremophilic locations are of great interest and have attracted substantial attention. *Microbacterium* strains have been found in various extremophilic locations, including Antarctic habitats [26], the Atacama Desert [27], sediment of the Indian Ocean [28], uranium-rich soil [29] and salty soil [30]. To be active in these extremophilic locations, enzymes from local *Microbacterium* strains may have to evolve. To the best of our knowledge, although some pioneering studies have revealed the important functions of some enzymes from *Microbacterium* strains in recent years [31–36], few of these enzymes were found to have adapted to extremophilic locations [32]. Furthermore, we found that all the GlcNAcases from *Microbacterium* strains were annotated based on *in silico* analysis without biochemical characterization. Therefore, the study may be the first to report an experimentally characterized GlcNAcase from *Microbacterium*.

Drastic inhibition by Ag^+^ and Hg^2+^ appears to be the common property of GlcNAcases, including HJ5Nag, the GH 20 GlcNAcases from *Paenibacillus* [25] and *V. furnissii* [37], the GH 3 GlcNAcases from *R. miehei* [38] and *Sphingobacterium* sp. [18], the GH 84 GlcNAcase from *C. paraputrificum* [39] and the GlcNAcase from *S. cerradoensis* [40]. Ag^+^ and Hg^2+^ have irreversible effects on sulfhydryl groups, and Hg^2+^ can also oxidize indole rings. Therefore, cysteine or tryptophan residues may be involved in the catalytic activity of GH 20 GlcNAcases. Sequence analysis revealed that W365, W430 and W465 of HJ5Nag might affect ligand binding. As shown in Fig. 1, the conserved W491 may also be essential for the activity of HJ5Nag. SDS is an inhibitor of GlcNAcases, in addition to Ag^+^ and Hg^2+^. All the GlcNAcases from *Microbacterium* sp. in this study, as well as *R. miehei* [38], *Sphingobacterium* sp. [18] and *L. casei* [41], are strongly inhibited by SDS. Purified rHJ5Nag showed peak activity at pH 6.0 and 45 °C. These properties are the same or similar to those of many GlcNAcases. For example, the GH 20 GlcNAcase from *S. thermoviolaceus* presents maximum activity at pH 6.0 and 50 °C [42], the GH 20 GlcNAcase from *H. latex* presents maximum activity at pH 6.0 and 45–50 °C [43], the GH 3 GlcNAcase from *R. miehei* presents maximum activity at pH 6.5 and 50 °C [38], and the GH 84 GlcNAcase from *Clostridium paraputrificum* presents maximum activity at pH 6.5 and 50 °C [39]. Furthermore, the molecular mass of HJ5Nag (55.9 kDa) is similar to that of some GlcNAcases, such as the GH 20 GlcNAcases from a soil-derived uncultured organism (53.4 kDa) [44], *C. fimi* (54.2 kDa) [45], *Paenibacillus* sp. (57.5 kDa) [25] and *S. thermoviolaceus* (58.3 kDa) [46], and the GH 3 GlcNAcase from *S. thermophilum* (55.9 kDa) [47].

Purified rHJ5Nag exhibited good salt tolerance, as the addition of most salts had little to no effect on the enzyme activity, especially CuSO_4_ and NaCl. Most GlcNAcases reported to date are strongly inhibited by Cu^2+^, such as the GH 20 GlcNAcases from *Paenibacillus* [25], *A. caviae* [48] and *L. casei* [41], the GH 3 GlcNAcase from *R. miehei* [38], the GH 84 GlcNAcase from *C. paraputrificum* [39] and the GlcNAcase from *S. cerradoensis* [40]. Furthermore, the GH 20 GlcNAcases from *Paenibacillus* are strongly inhibited by Ni^2+^ [25]; the GH20 GlcNAcase from *A. caviae* is strongly inhibited by Mn^2+^ [48]; the GH20 GlcNAcase from *T. reesei* is inhibited by Fe^3+^ [49]; the GH 84 GlcNAcase from *C. paraputrificum* is inhibited by Ni^2+^, Co^2+^, Mn^2+^, Zn^2+^ and Fe^3+^ [39]; and the GH 3 GlcNAcase from *R. miehei* is inhibited by Ni^2+^, Co^2+^, Zn^2+^ and EDTA [38]. Salt tolerance has attracted considerable attention because various salts are present or used in industries [14, 16, 18, 50–60], such as various marine materials and foods in China, with NaCl contents of 3.5–20.0% (w/w) [16, 18]. In addition, material processing and fermentation with a high concentration of NaCl is helpful to reduce the overall cost because sterilization is unnecessary [61].

To reveal the potential catalytic adaptations of rHJ5Nag to high concentrations of salt, sequence and structure comparisons to its homologs were performed. (I). Similar to the results from other salt-tolerant enzymes [50, 51, 53–59], statistical analysis of the amino acid residues showed that HJ5Nag presented higher proportions of acidic D and E along with A and G but lower proportions of C and K than its homologs (Table 1). Carboxyl groups from acidic amino acid residues can bind more water molecules and cations than other groups from other amino acid residues, especially K, and thus enable the protein to form a particle stabilized by cooperative hydrated ion networks [57, 58]. Hydrophobicity must be finely balanced to maintain a protein’s flexibility and stability [62]. High NaCl concentrations strengthen hydrophobic interactions, which can affect protein folding [62]. Salt-tolerant proteins are thought to reduce the size of hydrophobic residues to compensate for the increased hydrophobic effect caused by the high salt concentration [58]. The high proportion of A and G, which are small hydrophobic residues [57], is compatible with that function. In addition, a low abundance of C might give a protein more flexibility in high salt environments due to the decrease in rigid disulphide bridges [56]. (II). At the level of the secondary structure, a higher percentage of random coil structures at the expense of α-helices is observed for salt-tolerant proteins [56, 58], including HJ5Nag (Table 2). Greater protein rigidity is usually accompanied by a higher percentage of α-helix structures, while the opposite is observed for a higher percentage of random coils. (III). At the level of the tertiary structure, an excess of negatively charged surfaces is a typical property of salt-tolerant proteins [50, 51, 53, 56–59, 62, 63]. The increase in negative charge on the surface can counteract the lower dielectric constant at high salinity and improve the ability of enzymes to bind water and salt ions, thus improving protein solubility [56–58].

Most characterized GlcNAcases, including GH 3, 20 and 84 members, show specific activities or *V*_max_ of lower than 1000 μmol min^−1^ mg^−1^ towards *p*NPGlcNAc, as summarized in Table 3. In this study, rHJ5Nag exhibited a specificity activity of 1773.1 ± 1.1 μmol min^−1^ mg^−1^ and a *V*_max_ of 3097 ± 124 μmol min^−1^ mg^−1^ towards *p*NPGlcNAc. Furthermore, rHJ5Nag showed a specificity activity of 481.4 ± 2.3 μmol min^−1^ mg^−1^ towards GlcNAc_2_, which is much higher than the values from other GlcNAcases recorded in the literature, such as the values from *Sphingobacterium* sp. (0.3 μmol min^−1^ mg^−1^) [18], *T. spiralis* (0.79 nmol min^−1^ mg^−1^) [64], *R. miehei* (1.06 μmol min^−1^ mg^−1^) [38], *V. alginolyticus* (18.1 μmol min^−1^ mg^−1^) [65] and *S. thermophilum* (76.8 μmol min^−1^ mg^−1^) [47]. As discussed above, the structure of HJ5Nag may be more flexible because it has higher proportions of A, G and random coil structures but lower proportions of C and α-helix structures than its homologs. Furthermore, HJ5Nag has a higher proportion of D and E, which are not only negatively charged but also flexible residues. High B-factor values, which are believed to provide high structural flexibility, are usually observed for D and E. The high flexibility is presumed to be a factor in the high activity of the GH 20 GlcNAcase rHJ5Nag.

Although the synergistic action of chitinases and GlcNAcases for complete enzymatic degradation of chitin is attractive, there are few reports [18, 66, 67]. This study may be the first to report a synergistic effect between a chitinase and a GH 20 GlcNAcase. Significant synergy was observed between the commercial CtnSg and rHJ5Nag for chitin degradation, indicating that rHJ5Nag has great potential in the bioconversion of chitin waste. The tolerance of rHJ5Nag to GlcNAc may be one contributor to the significant synergy because GlcNAc can inhibit GlcNAcases and result in the accumulation of chitooligosaccharides, which can inhibit chitinases [38]. The GlcNAcases from *V. furnissii*, *Trichoderma harzianum*, *A. oryzae*, *S. frugiperda*, *R. miehei* and *T. spiralis* were competitively or noncompetitively inhibited by GlcNAc, with *K*_i_ values of 0.21 [68], 0.21 [69], 1.6 [13], 2.0 [70], 9.68 [38] and 15.75 mM [64], respectively. In this study, rHJ5Nag showed relatively high GlcNAc tolerance, with a *K*_i_ of 14.59 mM.

## Conclusions

A GH 20 GlcNAcase was isolated from *Microbacterium* sp. HJ5 harboured in the saline soil of an abandoned salt mine. Biochemical characterization revealed that the GlcNAcase had novel salt–GlcNAc tolerance and high activity. These characteristics suggest that the enzyme has versatile potential in biotechnological applications, such as bioconversion of chitin waste and processing of marine materials and saline foods. Molecular characterization provides an understanding of the molecular–function relationships for the salt tolerance and the high activity of the GH 20 GlcNAcase.

## Additional file

Additional file 1: Figure S1. The phylogenetic tree constructed on the basis of the 16S rDNA sequences of various genera of *Microbacteriaceae*. **Figure S2.** Structures of HJ5Nag and the GlcNAcase from *H. sapiens*. **Figure S3.** SDS–PAGE analysis of rHJ5Nag. **Figure S4.** MALDI–TOF MS spectrum of the purified protein. *hj5Nag* (KX400857). HJ5Nag translated from *hj5Nag. Microbacterium* sp. HJ5 16S rDNA (KX400858). (DOC 2860 kb)

## Abbreviations

A: Alanine
Buffer A: 20 mM Tris–HCl, 0.5 M NaCl, 10% (w/v) glycerol, pH 7.2
C: Cysteine
D: Aspartic acid
DNS: 3,5-dinitrosalicylic acid
*DS*: Degree of synergy
E: Glutamic acid
G: Glycine
GH: Glycoside hydrolase
GlcNAc: N-acetylglucosamine
GlcNAc_2_: N,N'-diacetyl chitobiose
GlcNAc_4_: N,N',N'',N'''-tetraacetyl chitotetraose
GlcNAcases: β-N-acetylglucosaminidases
HJ5Nag: The GlcNAcase from *Microbacterium* sp. HJ5
*hj5Nag*: The GlcNAcase-encoding gene from *Microbacterium* sp. HJ5
IPTG: Isopropyl-β-d-1-thiogalactopyranoside
K: Lysine
MALDI-TOF MS: Matrix-assisted laser desorption/ionization time-of-flight mass spectrometry
*p*NP: *p*-nitrophenol
*p*NPAra: *p*-nitrophenyl-α-l-arabinofuranoside
*p*NPGal: *p*-nitrophenyl α-d-galactopyranoside
*p*NPGalNAc: *p*-nitrophenyl β-N-acetylgalactosaminide
*p*NPGlc: *p*-nitrophenyl β-d-glucopyranoside
*p*NPGlcNAc: *p*-nitrophenyl β-N-acetylglucosaminide
*p*NPXyl: *p*-nitrophenyl-β-d-xylopyranoside
rHJ5Nag: The recombinant HJ5Nag
*RS*_CtnSg_: The amount of reducing sugars released by CtnSg from colloidal chitin without the addition of rHJ5Nag
*RS*_rHJ5Nag_: The amount of reducing sugars released by rHJ5Nag from colloidal chitin without the addition of CtnSg
*RS*_rHJ5NagCtnSg_: The amount of reducing sugars released from simultaneous or sequential enzyme reactions when determining the synergistic action
SDS-PAGE: Sodium dodecyl sulphate-polyacrylamide gel electrophoresis
TLC: Thin-layer chromatography

## References

[1] Litzinger S, Duckworth A, Nitzsche K, Risinger C, Wittmann V, Mayer C (2010). Muropeptide rescue in *Bacillus subtilis* involves sequential hydrolysis by β-N-acetylglucosaminidase and N-acetylmuramyl-L-alanine amidase. J Bacteriol.

[2] Patil RS, Ghormade V, Deshpande MV (2000). Chitinolytic enzymes: an exploration. Enzyme Microb Tech.

[3] Park JK, Kim WJ, Park YI (2011). Purification and characterization of an exo-type β-N-acetylglucosaminidase from *Pseudomonas fluorescens* JK-0412. J Appl Microbiol.

[4] Cody RM, Davis ND, Lin J, Shaw D (1990). Screening microorganisms for chitin hydrolysis and production of ethanol from amino sugars. Biomass.

[5] Vyas P, Deshpande M (1991). Enzymatic hydrolysis of chitin by *Myrothecium verrucaria* chitinase complex and its utilization to produce SCP. J Gen Appl Microbiol.

[6] Salvatore S, Heuschkel R, Tomlin S, Davies SE, Edwards S, Walker-Smith JA (2000). A pilot study of N-acetyl glucosamine, a nutritional substrate for glycosaminoglycan synthesis, in paediatric chronic inflammatory bowel disease. Aliment Pharm Ther.

[7] Shikhman AR, Amiel D, D’Lima D, Hwang SB, Hu C, Xu A (2005). Chondroprotective activity of N-acetylglucosamine in rabbits with experimental osteoarthritis. Ann Rheum Dis.

[8] Suzuki K, Mikami T, Okawa Y, Tokoro A, Suzuki S, Suzuki M (1986). Antitumor effect of hexa-N-acetylchitohexaose and chitohexaose. Carbohyd Res.

[9] Herlihey FA, Moynihan PJ, Clarke AJ (2014). The essential protein for bacterial flagella formation FlgJ functions as a β-N-acetylglucosaminidase. J Biol Chem.

[10] Okada S, Obrien JS (1969). Tay–Sachs disease: generalized absence of a β-D-N-acetylhexosaminidase component. Science.

[11] Li HZ, Morimoto K, Katagiri N, Kimura T, Sakka K, Lun S (2002). A novel β-N-acetylglucosaminidase of *Clostridium paraputrificum* M-21 with high activity on chitobiose. Appl Microbiol Biot.

[12] Matsuo I, Kim S, Yamamoto Y, Ajisaka K, Maruyama J, Nakajima H (2003). Cloning and overexpression of β-N-acetylglucosaminidase encoding gene *nagA* from *Aspergillus oryzae* and enzyme-catalyzed synthesis of human milk oligosaccharide. Biosci Biotech Bioch.

[13] Rajnochova E, Dvorakova J, Hunkova Z, Kren V (1997). Reverse hydrolysis catalysed by β-N-acetylhexosaminidase from *Aspergillus oryzae*. Biotechnol Lett.

[14] Zhou JP, Gao YJ, Dong YY, Tang XH, Li JJ, Xu B (2012). A novel xylanase with tolerance to ethanol, salt, protease, SDS, heat, and alkali from actinomycete *Lechevalieria* sp. HJ3. J Ind Microbiol Biot.

[15] Zhou JP, He LM, Gao YJ, Han NY, Zhang R, Wu Q (2016). Characterization of a novel low-temperature-active, alkaline and sucrose-tolerant invertase. Sci Rep.

[16] Zhou JP, Liu Y, Lu Q, Zhang R, Wu Q, Li CY (2016). Characterization of a glycoside hydrolase family 27 α-galactosidase from *Pontibacter* reveals its novel salt–protease tolerance and transglycosylation activity. J Agr Food Chem.

[17] Zhou JP, Lu Q, Zhang R, Wang YY, Wu Q, Li JJ (2016). Characterization of two glycoside hydrolase family 36 α-galactosidases: novel transglycosylation activity, lead–zinc tolerance, alkaline and multiple pH optima, and low-temperature activity. Food Chem.

[18] Zhou JP, Song ZF, Zhang R, Ding LM, Wu Q, Li JJ (2016). Characterization of a NaCl-tolerant β-N-acetylglucosaminidase from *Sphingobacterium* sp. HWLB1. Extremophiles.

[19] Zhou JP, Shen JD, Zhang R, Tang XH, Li JJ, Xu B (2015). Molecular and biochemical characterization of a novel multidomain xylanase from *Arthrobacter* sp. GN16 isolated from the feces of *Grus nigricollis*. Appl Biochem Biotech.

[20] Robert X, Gouet P (2014). Deciphering key features in protein structures with the new ENDscript server. Nucleic Acids Res.

[21] Willard L, Ranjan A, Zhang HY, Monzavi H, Boyko RF, Sykes BD (2003). VADAR: a web server for quantitative evaluation of protein structure quality. Nucleic Acids Res.

[22] Meux E, Morel M, Lamant T, Gerardin P, Jacquot JP, Dumarcay S (2013). New substrates and activity of *Phanerochaete chrysosporium* Omega glutathione transferases. Biochimie.

[23] Tews I, Perrakis A, Oppenheim A, Dauter Z, Wilson KS, Vorgias CE (1996). Bacterial chitobiase structure provides insight into catalytic mechanism and the basis of Tay–Sachs disease. Nat Struct Mol Biol.

[24] Lemieux MJ, Mark BL, Cherney MM, Withers SG, Mahuran DJ, James MNG (2006). Crystallographic structure of human β-hexosaminidase A: interpretation of Tay–Sachs mutations and loss of G(M2) ganglioside hydrolysis. J Mol Biol.

[25] Sumida T, Ishii R, Yanagisawa T, Yokoyama S, Ito M (2009). Molecular cloning and crystal structural analysis of a novel β-N-acetylhexosaminidase from *Paenibacillus* sp. TS12 capable of degrading glycosphingolipids. J Mol Biol.

[26] Han SR, Kim KH, Ahn DH, Park H, Oh TJ (2016). Complete genome sequence of carotenoid-producing *Microbacterium* sp. strain PAMC28756 isolated from an Antarctic lichen. J Biotechnol.

[27] Mandakovic D, Cabrera P, Pulgar R, Maldonado J, Aravena P, Latorre M (2015). Complete genome sequence of *Microbacterium* sp. CGR1, bacterium tolerant to wide abiotic conditions isolated from the Atacama Desert. J Biotechnol.

[28] Zhang YB, Ren HH, Zhang GY (2014). *Microbacterium hydrothermale* sp. nov., an actinobacterium isolated from hydrothermal sediment. Int J Syst Evol Micr.

[29] Mondani L, Piette L, Christen R, Bachar D, Berthomieu C, Chapon V (2013). *Microbacterium lemovicicum* sp. nov., a bacterium isolated from a natural uranium-rich soil. Int J Syst Evol Micr.

[30] Kook M, Son HM, Yi TH (2014). *Microbacterium kyungheense* sp. nov. and *Microbacterium jejuense* sp. nov., isolated from salty soil. Int J Syst Evol Micr.

[31] Valk V, Eeuwema W, Sarian FD, van der Kaaij RM, Dijkhuizen L (2015). Degradation of granular starch by the bacterium *Microbacterium aurum* strain B8.A involves a modular α-amylase enzyme system with FNIII and CBM25 domains. Appl Environ Microb.

[32] Lu J, Wu XD, Jiang YL, Cai XF, Huang LY, Yang YB (2014). An extremophile *Microbacterium* strain and its protease production under alkaline conditions. J Basic Microbiol.

[33] Isotani K, Kurokawa J, Suzuki F, Nomoto S, Negishi T, Matsuda M (2013). Gene cloning and characterization of two NADH-dependent 3-quinuclidinone reductases from *Microbacterium luteolum* JCM 9174. Appl Environ Microb.

[34] Kim DW, Feng JH, Chen HZ, Kweon O, Gao Y, Yu LR (2013). Identification of the enzyme responsible for N-acetylation of norfloxacin by *Microbacterium* sp. strain 4 N2-2. Appl Environ Microb.

[35] Ohta Y, Hatada Y, Hidaka Y, Shimane Y, Usui K, Ito T (2014). Enhancing thermostability and the structural characterization of *Microbacterium saccharophilum* K-1 β-fructofuranosidase. Appl Microbiol Biot.

[36] Wang JJ, Zhu YX, Zhao GG, Zhu JG, Wu S (2015). Characterization of a recombinant (+)-γ-lactamase from *Microbacterium hydrocarbonoxydans* which provides evidence that two enantiocomplementary γ-lactamases are in the strain. Appl Microbiol Biot.

[37] Keyhani NO, Roseman S (1996). The chitin catabolic cascade in the marine bacterium *Vibrio furnissii*—molecular cloning, isolation, and characterization of a periplasmic β-N-acetylglucosaminidase. J Biol Chem.

[38] Yang SQ, Song S, Yan QJ, Fu X, Jiang ZQ, Yang XB (2014). Biochemical characterization of the first fungal glycoside hydrolyase family 3 β-N-acetylglucosaminidase from *Rhizomucor miehei*. J Agr Food Chem.

[39] Li HZ, Morimoto K, Kimura T, Sakka K, Ohmiya K (2003). A new type of β-N-acetylglucosaminidase from hydrogen-producing *Clostridium paraputrificum* M-21. J Biosci Bioeng.

[40] da Silva Junior Sobrinho I, Bataus LAM, Maitan VR, Ulhoa CJ (2005). Purification and properties of an N-acetylglucosaminidase from *Streptomyces cerradoensis*. Biotechnol Lett.

[41] Senba M, Kashige N, Nakashima K, Miake F, Watanabe K (2000). Cloning of the gene of β-N-acetylglucosaminidase from *Lactobacillus casei* ATCC 27092 and characterization of the enzyme espressed in *Escherichia coli*. Biol Pharm Bull.

[42] Kubota T, Miyamoto K, Yasuda M, Inamori Y, Tsujibo H (2004). Molecular characterization of an intracellular β-N-acetylglucosaminidase involved in the chitin degradation system of *Streptomyces thermoviolaceus* OPC-520. Biosci Biotech Bioch.

[43] Sukprasirt P, Wititsuwannakul R (2014). A chitinolytic endochitinase and β-N-acetylglucosaminidase-based system from *Hevea latex* in generating N-acetylglucosamine from chitin. Phytochemistry.

[44] Nyffenegger C, Nordvang RT, Zeuner B, Lezyk M, Difilippo E, Logtenberg MJ (2015). Backbone structures in human milk oligosaccharides: trans-glycosylation by metagenomic β-N-acetylhexosaminidases. Appl Microbiol Biot.

[45] Mayer C, Vocadlo DJ, Mah M, Rupitz K, Stoll D, Warren RAJ (2006). Characterization of a β-N-acetylhexosaminidase and a β-N-acetylglucosaminidase/β-glucosidase from *Cellulomonas fimi*. FEBS J.

[46] Tsujibo H, Hatano N, Mikami T, Izumizawa Y, Miyamoto K, Inamori Y (1998). Cloning, characterization and expression of β-N-acetylglucosaminidase gene from *Streptomyces thermoviolaceus* OPC-520. BBA-Gen Subjects.

[47] Ogawa M, Kitagawa M, Tanaka H, Ueda K, Watsuji T, Beppu T (2006). A β-N-acetylhexosaminidase from *Symbiobacterium thermophilum*; gene cloning, overexpression, purification and characterization. Enzyme Microb Tech.

[48] Lin H, Xiao X, Zeng X, Wang FP (2006). Expression, characterization and mutagenesis of the gene encoding β-N-acetylglucosaminidase from *Aeromonas caviae* CB101. Enzyme Microb Tech.

[49] Chen F, Chen XZ, Qin LN, Tao Y, Dong ZY (2015). Characterization and homologous overexpression of an N-acetylglucosaminidase Nag1 from *Trichoderma reesei*. Biochem Bioph Res Co.

[50] Liu XS, Huang ZQ, Zhang XN, Shao ZZ, Liu ZD (2014). Cloning, expression and characterization of a novel cold-active and halophilic xylanase from *Zunongwangia profunda*. Extremophiles.

[51] Yamaguchi R, Tokunaga H, Ishibashi M, Arakawa T, Tokunaga M (2011). Salt-dependent thermo-reversible α-amylase: cloning and characterization of halophilic α-amylase from moderately halophilic bacterium. *Kocuria varians*. Appl Microbiol Biot.

[52] Wang GZ, Wang QH, Lin XJ, Ng TB, Yan RX, Lin J (2016). A novel cold-adapted and highly salt-tolerant esterase from *Alkalibacterium* sp. SL3 from the sediment of a soda lake. Sci Rep.

[53] Premkumar L, Greenblatt HM, Bageshwar UK, Savchenko T, Gokhman I, Sussman JL (2005). Three-dimensional structure of a halotolerant algal carbonic anhydrase predicts halotolerance of a mammalian homolog. Proc Natl Acad Sci U S A.

[54] Shen JD, Zhang R, Li JJ, Tang XH, Li RX, Wang M (2015). Characterization of an exo-inulinase from *Arthrobacter*: a novel NaCl-tolerant exo-inulinase with high molecular mass. Bioengineered.

[55] Zhou JP, Peng MZ, Zhang R, Li JJ, Tang XH, Xu B (2015). Characterization of *Sphingomonas* sp. JB13 exo-inulinase: a novel detergent-, salt-, and protease-tolerant exo-inulinase. Extremophiles.

[56] Paul S, Bag SK, Das S, Harvill ET, Dutta C (2008). Molecular signature of hypersaline adaptation: insights from genome and proteome composition of halophilic prokaryotes. Genome Biol.

[57] Madern D, Ebel C, Zaccai G (2000). Halophilic adaptation of enzymes. Extremophiles.

[58] Warden AC, Williams M, Peat TS, Seabrook SA, Newman J, Dojchinov G (2015). Rational engineering of a mesohalophilic carbonic anhydrase to an extreme halotolerant biocatalyst. Nat Commun.

[59] Qin YJ, Huang ZQ, Liu ZD (2014). A novel cold-active and salt-tolerant α-amylase from marine bacterium *Zunongwangia profunda*: molecular cloning, heterologous expression and biochemical characterization. Extremophiles.

[60] Shi RR, Li ZM, Ye Q, Xu JH, Liu Y (2013). Heterologous expression and characterization of a novel thermo-halotolerant endoglucanase Cel5H from *Dictyoglomus thermophilum*. Bioresource Technol.

[61] Margesin R, Schinner F (2001). Potential of halotolerant and halophilic microorganisms for biotechnology. Extremophiles.

[62] Siglioccolo A, Paiardini A, Piscitelli M, Pascarella S (2011). Structural adaptation of extreme halophilic proteins through decrease of conserved hydrophobic contact surface. BMC Struct Biol.

[63] Wu GJ, Wu GB, Zhan T, Shao ZZ, Liu ZD (2013). Characterization of a cold-adapted and salt-tolerant esterase from a psychrotrophic bacterium *Psychrobacter pacificensis*. Extremophiles.

[64] Bruce AF, Gounaris K (2006). Characterisation of a secreted N-acetyl-β-hexosaminidase from *Trichinella spiralis*. Mol Biochem Parasit.

[65] Ohishi K, Murase K, Etoh H (1999). Purification and properties of β-N-acetylglucosaminidase from *Vibrio alginolyticus* H-8. J Biosci Bioeng.

[66] Fukamizo T, Kramer KJ (1985). Mechanism of chitin oligosaccharide hydrolysis by the binary enzyme chitinase system in insect moulting fluid. Insect Biochem.

[67] Suzuki K, Sugawara N, Suzuki M, Uchiyama T, Katouno F, Nikaidou N (2002). Chitinases A, B, and C1 of *Serratia marcescens* 2170 produced by recombinant *Escherichia coli*: enzymatic properties and synergism on chitin degradation. Biosci Biotech Bioch.

[68] Chitlaru E, Roseman S (1996). Molecular cloning and characterization of a novel β-N-acetyl-D-glucosaminidase from *Vibrio furnissii*. J Biol Chem.

[69] Nieder V, Kutzer M, Kren V, Gallego RG, Kamerling JP, Elling L (2004). Screening and characterization of β-N-acetylhexosaminidases for the synthesis of nucleotide-activated disaccharides. Enzyme Microb Tech.

[70] Tomiya N, Narang S, Park J, Abdul-Rahman B, Choi O, Singh S (2006). Purification, characterization, and cloning of a *Spodoptera frugiperda* Sf9 β-N-acetylhexosaminidase that hydrolyzes terminal N-acetylglucosamine on the N-glycan core. J Biol Chem.

[71] Suginta W, Chuenark D, Mizuhara M, Fukamizo T (2010). Novel β-N-acetylglucosaminidases from *Vibrio harveyi* 650: cloning, expression, enzymatic properties, and subsite identification. BMC Biochem.

[72] Liu TA, Zhang HT, Liu FY, Wu QY, Shen X, Yang Q (2011). Structural determinants of an insect β-N-acetyl-D-hexosaminidase specialized as a chitinolytic enzyme. J Biol Chem.

[73] Lan XQ, Ozawa N, Nishiwaki N, Kodaira R, Okazaki M, Shimosaka M (2004). Purification, cloning, and sequence analysis of β-N-acetylglucosaminidase from the chitinolytic bacterium *Aeromonas hydrophila* strain SUWA-9. Biosci Biotech Bioch.

[74] Mark BL, Wasney GA, Salo TJS, Khan AR, Cao ZM, Robbins PW (1998). Structural and functional characterization of *Streptomyces plicatus* β-N-acetylhexosaminidase by comparative molecular modeling and site-directed mutagenesis. J Biol Chem.

[75] Ueda M, Fujita Y, Kawaguchi T, Arai M (2000). Cloning, nucleotide sequence and expression of the β-N-acetylglucosaminidase gene from *Aeromonas* sp. no. 10S-24. J Biosci Bioeng.

[76] Lan XQ, Zhang X, Kodaira R, Zhou Z, Shimosaka M (2008). Gene cloning, expression, and characterization of a second β-N-acetylglucosaminidase from the chitinolytic bacterium *Aeromonas hydrophila* strain SUWA-9. Biosci Biotech Bioch.

[77] Riekenberg S, Flockenhaus B, Vahrmann A, Muller MCM, Leippe M, Kiess M (2004). The β-N-acetylhexosaminidase of *Entamoeba histolytica* is composed of two homologous chains and has been localized to cytoplasmic granules. Mol Biochem Parasit.

[78] Konno N, Takahashi H, Nakajima M, Takeda T, Sakamoto Y (2012). Characterization of β-N-acetylhexosaminidase (LeHex20A), a member of glycoside hydrolase family 20, from *Lentinula edodes* (shiitake mushroom). AMB Express.

[79] Tsujibo H, Fujimoto K, Tanno H, Miyamoto K, Kimura Y, Imada C (1995). Molecular cloning of the gene which encodes β-N-acetylglucosaminidase from a marine bacterium, *Alteromonas* sp. strain O-7. Appl Environ Microb.

[80] Tsujibo H, Miyamoto K, Yoshimura M, Takata M, Miyamoto J, Inamori Y (2002). Molecular cloning of the gene encoding a novel β-N-acetylhexosaminidase from a marine bacterium, *Alteromonas* sp. strain O-7, and characterization of the cloned enzyme. Biosci Biotech Bioch.

[81] Slamova K, Kulik N, Fiala M, Krejzova-Hofmeisterova J, Ettrich R, Kren V (2014). Expression, characterization and homology modeling of a novel eukaryotic GH84 β-N-acetylglucosaminidase from *Penicillium chrysogenum*. Protein Expres Purif.

[82] Meekrathok P, Suginta W (2016). Probing the catalytic mechanism of *Vibrio harveyi* GH20 β-N-acetylglucosaminidase by chemical rescue. PLoS One.

[83] Tsujibo H, Hatano N, Mikami T, Hirasawa A, Miyamoto K, Inamori Y (1998). A novel β-N-acetylglucosaminidase from *Streptomyces thermoviolaceus* OPC-520: gene cloning, expression, and assignment to family 3 of the glycosyl hydrolases. Appl Environ Microb.

[84] Choi KH, Seo JY, Park KM, Park CS, Cha J (2009). Characterization of glycosyl hydrolase family 3 β-N-acetylglucosaminidases from *Thermotoga maritima* and *Thermotoga neapolitana*. J Biosci Bioeng.

[85] Matsuo Y, Kurita M, Park JK, Tanaka K, Nakagawa T, Kawamukai M (1999). Purification, characterization and gene analysis of N-acetylglucosaminidase from *Enterobacter* sp. G-1. Biosci Biotech Bioch.

[86] O’Connell E, Murray P, Piggott C, Hennequart F, Tuohy M (2008). Purification and characterization of a N-acetylglucosaminidase produced by *Talaromyces emersonii* during growth on algal fucoidan. J Appl Phycol.

