# Supplementary material for: Distinctive molecular and biochemical characteristics of a glycoside hydrolase family 20 β-N-acetylglucosaminidase and salt tolerance
Source: BMC Biotechnol. 2017 Apr 11;17:37. doi: 10.1186/s12896-017-0358-1 (PMC5387316; doi:10.1186/s12896-017-0358-1)
Supplement: Additional file 1: Figure S1. — The phylogenetic tree constructed on the basis of the 16S rDNA sequences of various genera of Microbacteriaceae. Figure S2. Structures of HJ5Nag and the GlcNAcase from H. sapiens. Figure S3. SDS–PAGE analysis of rHJ5Nag. Figure S4. MALDI–TOF MS spectrum of the purified protein. hj5Nag (KX400857). HJ5Nag translated from hj5Nag. Microbacterium sp. HJ5 16S rDNA (KX400858). (DOC 2860 kb) [file 12896_2017_358_MOESM1_ESM.doc]

**Distinctive molecular and biochemical characteristics of a glycoside hydrolase family 20 β-N-acetylglucosaminidase** **and salt tolerance**

Junpei Zhou^1,2,3,4†^, Zhifeng Song^2†^, Rui Zhang^1,2,3,4^, Rui Liu^2^, Qian Wu^1,2,3,4^, Junjun Li^1,2,3,4^, Xianghua Tang^1,2,3,4^, Bo Xu^1,2,3,4^, Junmei Ding^1,2,3,4^, Nanyu Han^1,2,3,4^, Zunxi Huang1,2,3,4 [[1]](#footnote-2)*

^1^Engineering Research Center of Sustainable Development and Utilization of Biomass Energy, Ministry of Education, Yunnan Normal University, Kunming, 650500, People’s Republic of China

^2^College of Life Sciences, Yunnan Normal University, Kunming, 650500, People’s Republic of China

^3^Key Laboratory of Yunnan for Biomass Energy and Biotechnology of Environment, Yunnan, Kunming, 650500, People’s Republic of China

^4^Key Laboratory of Enzyme Engineering, Yunnan Normal University, Kunming, 650500, People’s Republic of China

^†^J.Z. and Z.S. contributed equally to this work

**Fig. S1** The phylogenetic tree constructed on the basis of the 16S rDNA sequences of various genera of *Microbacteriaceae*.

Multiple 16S rDNA sequences alignment, distance matrice calculation (Kimura two-parameter model) and phylogenetic tree construction (neighbor-joining algorithm) were performed with MEGA [1]. Accession numbers are given in *parentheses*. The 16S rDNA sequence from HJ5 is shown in *bold* *type*. Bootstrap values (*n* = 1,000 replicates) are reported as percentages. The *scale bar* represents the number of changes per nucleotide position.

**Fig. S2** Structures of HJ5Nag and the GlcNAcase from *H. sapiens*.

The A-chain of the GlcNAcase from *H. sapiens* (PDB ID: 2GJX) is used.

**Fig. S3** SDS–PAGE analysis of rHJ5Nag.

Lanes: CK, cell extract of an induced negative transformant; S, cell extract of an induced positive transformant; M, protein molecular weight marker; P, purified rHJ5Nag

**Fig. S4** MALDI–TOF MS spectrum of the purified protein.

>*hj5Nag* (KX400857)

ATGAATCGTCGCCGAGGACGGGCCATCGCCGCCGCCACGGTGCTCGCCGCGTCGCTGGCGGGGTGCAGCCCCGCCGCCCAGAATGGAGACACTGTGCCCCTTCCCGCCGTCGTGCCCGCCCCCGCCGCCATCGAGCAGGCGACCGGCGCGCCGTTCCGCCTCGACGCGTCGACGCGGATCGAAGGAGAAGCGGATGCCGCGTCCGCCCTGTCCGCTCTCCTCGAAGCCCGCACCGGCCTGGCCCCCGCGACCGGGGGCGATGGTGCCGTGATCGCCCTCCGCATCGAGGGAGGCGGCCCGGCCGAGTCGTACGCGCTCACGGCGGACGAGGCATCCGTCACCGTCACGGGCGCCGACGCTGCGGGACTCTTCTACGGCGTGCAGACCCTCGGCCAGCTGCTCGCCCGCGACGGCGACGCGTGGGTCGTGCCGGCCGTCTCGATCGAGGACGCCCCGCGCTTCGCCTACCGCGGCGTCATGCTCGACGTCGCCCGCCACTTCCACCCCGTCGAGACGGTCAAGGCCTACATCGGTCACGCGGCGAGCCTCAAGCTCAACGCCCTGCACCTGCACCTCAGCGACGACCAGGGATGGCGCATCGAGCTGCACTCGCGGCCGGAGCTCACCGCGCTCGCCTCGTCGACGGCCGTCGGCGGCGACCCGGGCGGCTTCTACACGAAGGACGACTACCGCGAGATCGTCGAGTATGCGGCATCCCGTCACATGATCGTCGTGCCCGAGATCGACATGCCGAGCCACACCCACGCGATCGGACTCGCCTACCCCGAGCTCGCGGAGGAGCCGGTCATCACCGACCCGATGCGCGAGACCGCGGCGGCCACCGGCGGTGCGCTGCCCGAGAGCGGCACGCCCTACCTGGGGATCGAGGTCGGGTTCTCGTCGCTGAAGATCCACGACGAGGCGACCTACGACTTCGCGGCCGACGTGTTCGGCGAGCTCGCGGGGATGACCCCCGGCCCGTACCTTCACCTGGGCGGCGACGAGGCGCACGGCACCGCCGAGGAGGACTTCGCCCTGTTCGTGTCGCGCGTGAGCACGATCATCGCCGACCTCGGCAAGACGCCCGTCGCGTGGCACGAGGCGGGCGACGCGGGGGGCCTCGCCGGGGCGACCGTGGGGCAGTACTGGGGCTACGTGACGCCGACCGACGGCATGGACGACCGTGCGCGGGGATTCGTCTCCAACGGCGGGCAGCTGATCCTGTCGCCCGCCGACGCGATCTACCTCGACATGAAGTACCCGACCGGTCCCGACCTCGGCCTGTCGTGGGCCAACGGCCCGACCAGCGTGCAGCGCGCATACGACTGGGAGCCGTCCACGGTGATCCCGGGCATCGACGATGCCGACATCCTCGGCGTCGAGGCACCCCTCTGGAGCGAGACGCTGCGATCGCTCGACGACATCGAGACCATGGCCTTCCCGCGGATCGCCGCGGCCGCCGAGGCGGCATGGTCGCCCGCGACCGGGGCGAGCGATCTGCGCACGTGGGAGTCGTTCCGCGCCCGCGTCGGCGCGCTCGGCCCGCTGTGGACGAGCCTCGGCATCGGCTTCCACCCGAGCGGCGAGATCGACTGGGCCACCGAGTGA

>HJ5Nag translated from *hj5Nag*

MNRRRGRAIAAATVLAASLAGCSPAAQNGDTVPLPAVVPAPAAIEQATGAPFRLDASTRIEGEADAASALSALLEARTGLAPATGGDGAVIALRIEGGGPAESYALTADEASVTVTGADAAGLFYGVQTLGQLLARDGDAWVVPAVSIEDAPRFAYRGVMLDVARHFHPVETVKAYIGHAASLKLNALHLHLSDDQGWRIELHSRPELTALASSTAVGGDPGGFYTKDDYREIVEYAASRHMIVVPEIDMPSHTHAIGLAYPELAEEPVITDPMRETAAATGGALPESGTPYLGIEVGFSSLKIHDEATYDFAADVFGELAGMTPGPYLHLGGDEAHGTAEEDFALFVSRVSTIIADLGKTPVAWHEAGDAGGLAGATVGQYWGYVTPTDGMDDRARGFVSNGGQLILSPADAIYLDMKYPTGPDLGLSWANGPTSVQRAYDWEPSTVIPGIDDADILGVEAPLWSETLRSLDDIETMAFPRIAAAAEAAWSPATGASDLRTWESFRARVGALGPLWTSLGIGFHPSGEIDWATE*

>*Microbacterium* sp. HJ5 16S rDNA (KX400858)

atgcagtcgaacggtgaagccaagcttgcttggtggatcagtggcgaacgggtgagtaacacgtgagcaacctgccccggactctgggataagcgctggaaacggcgtctaatactggatacgagcttcagtcgcatggctaggagctggaaagattttttggtctgggatgggctcgcggcctatcagcttgttggtgaggtaatggctcaccaaggcgtcgacgggtagccggcctgagagggtgaccggccacactgggactgagacacggcccagactcctacgggaggcagcagtggggaatattgcacaatgggcggaagcctgatgcagcaacgccgcgtgagggatgacggccttcgggttgtaaacctcttttagcaaggaagaagcgaaagtgacggtacttgcagaaaaagcgccggctaactacgtgccagcagccgcggtaatacgtagggcgcaagcgttatccggaattattgggcgtaaagagctcgtaggcggtttgtcgcgtctgctgtgaaatcccgaggctcaacctcgggcctgcagtgggtacgggcagactagagtgcggtaggggagattggaattcctggtgtagcggtggaatgcgcagatatcaggaggaacaccgatggcgaaggcagatctctgggccgtaactgacgctgaggagcgaaagggtggggagcaaacaggcttagataccctggtagtccaccccgtaaacgttgggaactagttgtggggtccattccacggattccgtgacgcagctaacgcattaagttccccgcctggggagtacggccgcaaggctaaaactcaaaggaattgacggggacccgcacaagcggcggagcatgcggattaattcgatgcaacgcgaagaaccttaccaaggcttgacatacacgagaacgccctagaaatagggaactctttggacactcgtgaacaggtggtgcatggttgtcgtcagctcgtgtcgtgagatgttgggttaagtcccgcaacgagcgcaaccctcgttctatgttgccagcacgtaatggtgggaactcatgggatactgccggggtcaactcggaggaaggtggggatgacgtcaaatcatcatgccccttatgtcttgggcttcacgcatgctacaatggccggtacaaagggctgcaataccgtgaggtggagcgaatcccaaaaagccggtcccagttcggattgaggtctgcaactcgacctcatgaagtcggagtcgctagtaatcgcagatcagcaacgctgcggtgaatacgttcccgggtcttgtacacaccgcccgtcaagtcatgaaagtcggtaacacctgaagccggtggcccaatcc

**References**

1. Tamura K, Dudley J, Nei M, Kumar S. MEGA4: molecular evolutionary genetics analysis (MEGA) software version 4.0. Mol Biol Evol. 2007;24(8):1596–9.

1. ^*^ **Correspondence**: Zunxi Huang, College of Life Sciences, Yunnan Normal University, No. 768 Juxian Street, Chenggong, Kunming, Yunnan 650500, People’s Republic of China. Tel.: +86 871 65920830; Fax: +86 871 65920952; e-mail: huangzunxi@163.com [↑](#footnote-ref-2)
